# Mass Spectrometry Imaging of atherosclerosis-affine Gadofluorine following Magnetic Resonance Imaging

**DOI:** 10.1038/s41598-019-57075-6

**Published:** 2020-01-09

**Authors:** Fabian Lohöfer, Rebecca Buchholz, Almut Glinzer, Katharina Huber, Helena Haas, Georgios Kaissis, Annette Feuchtinger, Michaela Aichler, Peter B. Sporns, Carsten Höltke, Miriam Stölting, Franz Schilling, René M. Botnar, Melanie A. Kimm, Cornelius Faber, Axel K. Walch, Alma Zernecke, Uwe Karst, Moritz Wildgruber

**Affiliations:** 1 Department of Diagnostic and Interventional Radiology, School of Medicine & Klinikum rechts der Isar, Technical University of Munich, Munich, Germany; 2 Department for Analytical Chemistry, Westfälische Wilhelms-Universität, Münster, Germany; 3 Research Unit Analytical Pathology, German Research Center for Environmental Health, Helmholtz Zentrum München, Neuherberg, Germany; 4 Translational Research Imaging Center, Department of Clinical Radiology, Westfälische Wilhelms-Universität, Münster, Germany; 5 Department of Nuclear Medicine, School of Medicine & Klinikum rechts der Isar, Technical University of Munich, Munich, Germany; 6 School of Biomedical Engineering and Imaging Sciences, King’s College London, London, UK; 7 Institute for Experimental Biomedicine, Universitätsklinikum Würzburg, Würzburg, Germany; 8 DFG Cluster of Excellence EXC 1003 ‘Cells in Motion’, Münster, Germany

**Keywords:** Biosensors, Preclinical research

## Abstract

Molecular imaging of atherosclerosis by Magnetic Resonance Imaging (MRI) has been impaired by a lack of validation of the specific substrate responsible for the molecular imaging signal. We therefore aimed to investigate the additive value of mass spectrometry imaging (MSI) of atherosclerosis-affine Gadofluorine P for molecular MRI of atherosclerotic plaques. Atherosclerotic *Ldlr*^*−/−*^ mice were investigated by high-field MRI (7 T) at different time points following injection of atherosclerosis-affine Gadofluorine P as well as at different stages of atherosclerosis formation (4, 8, 16 and 20 weeks of HFD). At each imaging time point mice were immediately sacrificed after imaging and aortas were excised for mass spectrometry imaging: Matrix Assisted Laser Desorption Ionization (MALDI) Imaging and Laser Ablation – Inductively Coupled Plasma – Mass Spectrometry (LA-ICP-MS) imaging. Mass spectrometry imaging allowed to visualize the localization and measure the concentration of the MR imaging probe Gadofluorine P in plaque tissue *ex vivo* with high spatial resolution and thus adds novel and more target specific information to molecular MR imaging of atherosclerosis.

## Introduction

Visualization of atherosclerosis using MR imaging is an emerging tool to gain deeper and more dynamic insights into biological processes of atherosclerosis with a high spatial and temporal resolution as well as high sensitivity. An advantage of MR imaging is the ability to acquire anatomical, functional and biological information simultaneously. Noninvasive characterization and assessment of plaque burden, characterization of plaque features (molecular and anatomical) and monitoring of plaque progression are crucial to predict plaque rupture, which causes myocardial infarction or stroke.

Various molecular MRI approaches have tried to characterize specific aspects of atherosclerosis. Endothelial permeability, one of the key features of early vascular dysfunction, can be assessed by using albumin-affine gadolinium (Gd) chelates such as Gadofosveset trisodium^1,2^. Influx of inflammatory cells such as proinflammatory macrophages can be traced by using iron-oxide nanoparticles, which has been similarly successful both in mice and humans. Additionally, the complex process of vascular remodeling has been successfully investigated by targeting tropoelastin, elastin and other extracellular proteins by more or less specific gadolinium agents in mice and rabbits^3–5^. All approaches rely on alteration of the MR signal induced either by shortening T1 relaxation (in case of gadolinium) or shortening T2* (in case of iron-oxide nanoparticles), generated by accumulation of a protein or cell specific probe at the target site. Signal generation in MRI, however, is never specific for a singular cellular or molecular process as the signal is always a superposition of endogenous contrast generation and externally applied probes. Thus, T1 shortening in atherosclerosis imaging can be generated by accumulation of a gadolinium-based agent and/or intraplaque hemorrhage, with MRI being unable to separate both. Therefore, in *in vivo* MRI it cannot be defined to which degree a certain substrate e.g. a molecular contrast agent contributes to the actual imaging signal. Traditional methods such as immunohistochemistry have severe drawbacks as they only can detect biological plaque components but fail to visualize molecular MR agents specifically. Gadolinium concentration assessment in atherosclerotic plaques with inductively coupled plasma - mass spectroscopy (ICP-MS) provides accurate quantification, but lacks anatomic information^6^. An imaging technique which is able to detect lanthanoids such as Gd, and provide anatomical information is imaging mass cytometry (IMC). Lanthanoid-labelled antibodies can be used to stain defined structures in tissue thin sections^7^. This approach was recently used to detect Platin from cytostatics in tumor tissue^8^. The applicability for Gd-based contrast agents has to be proven since these molecules are highly water-soluble and IMC workflow contains washing and equilibration steps in aqueous solution. Mass spectrometry imaging of gadolinium chelates has been achieved by matrix-assisted laser desorption ionization imaging (MALDI MSI)^9^ and laser ablation - inductively coupled plasma - mass spectrometry (LA-ICP-MS)^10–12^. Mass spectrometry imaging has been used in the past years to provide comprehensive maps of lipid and protein distribution in experimental and clinical atherosclerosis down to a spatial resolution of 30 μm. These approaches allow for detailed analysis of key molecular alterations during atherosclerotic plaque development and potentially to detect therapy induced changes in the future^13–17^. With the advent of quantifying both, anatomy at a molecular level and similarly detecting dedicated elements we sought to investigate for the first time how mass spectrometry imaging can provide additional value to molecular MR imaging of atherosclerosis. We hypothesize that mass spectrometry imaging is able to visualize and quantify a molecular MR imaging agent within atherosclerotic plaques with high spatial resolution.

## Results

### Gadofluorine P accumulation in atherosclerotic plaques

**Figure 1:**
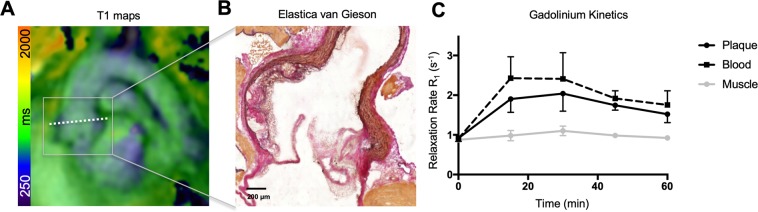
T1 mapping of Gadofluorine P accumulation in murine atherosclerosis. (**A**) Longitudinal Magnetic Resonance Imaging (in n = 3 mice) was performed after injection of 0.2 mmol/kg body weight Gadofluorine P in an oblique slice orientation for detection of atherosclerotic plaques at the aortic root. Overlay of T1 mapping and late gadolinium enhancement of a representative animal is shown. Additional T1 mapping was performed in axial slice orientation (shown as dotted line) for localization of atherosclerotic plaques in two dimensions. (**B**) Plaque formation was confirmed by histology (Elastica van Gieson staining). (**C**) Enhancement kinetics were investigated in *Ldlr*^*−/−*^ mice after 16 weeks on high-fat diet. R1 relaxation rates are shown over the period of 60 minutes following injection. Data are from continuous measurements are shown as Mean ± SEM, n = 3 data sets per time point.

To assess its’ potential to target atherosclerotic plaques, Gadofluorine P was injected in *Ldlr*^*−/−*^ mice (n = 3) after those have received a high-fat diet for 16 weeks. MR imaging was performed longitudinally pre and at 15, 30, 45 and 60 minutes post injection to determine the kinetics of accumulation within the atherosclerotic vessel wall. Imaging was performed in oblique and transverse slice orientation (Fig. 1A). Plaques were most prominent at the aortic root (Fig. 1B) which was therefore chosen as the target region. R1 values increased significantly after injection and peaked at 30 minutes post injection (p = 0.0036) within the atherosclerotic plaque with significantly higher R1 values compared to the adjacent myocardium (p < 0.0001) and decreased thereafter (Fig. 1C). A positive contrast between the atherosclerotic wall and adjacent blood and myocardium was present at all time points post injection. Due to the highest signal at 30 minutes post injection, subsequent MR image acquisition was performed always at 30 minutes post Gadofluorine P injection to assess progression of atherosclerosis at different time points of high-fat diet.

### Detection of Gadofluorine P in atherosclerotic plaques by MALDI MSI

**Figure 2:**
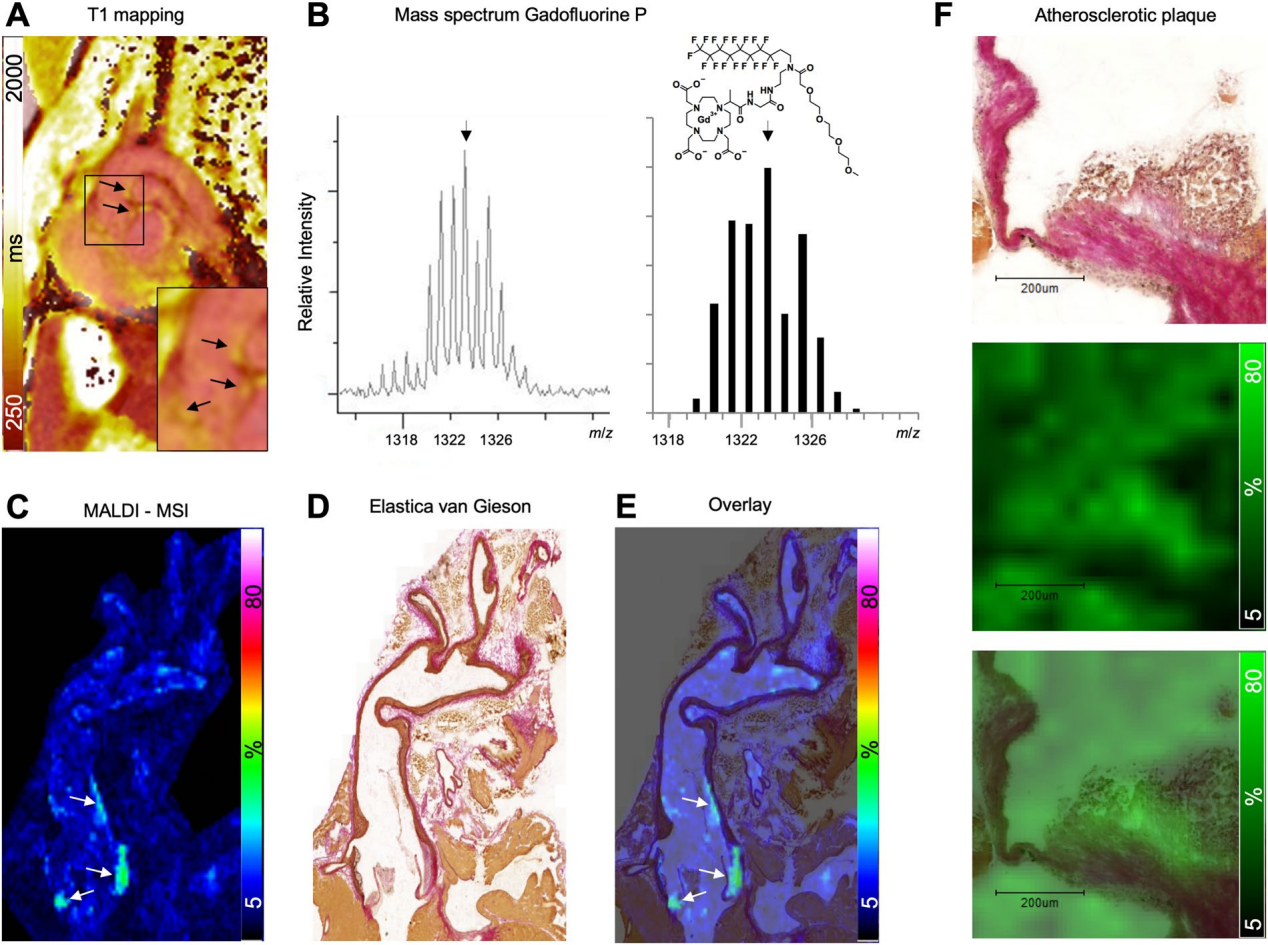
Assessment of Gadofluorine P accumulation by MALDI MSI. (**A**) Atherosclerotic plaques were detected by T1 mapping predominantly at the aortic root following Gadofluorine P injection. MR image shows overlay of T1 mapping and late gadolinium enhancement for better anatomical correlation. (**B**) Gadofluorine P can be detected by mass spectrometry based upon its monoisotopic peak of 1323 Da allowing its visualization by MALDI MSI. Left panel depicts the actual spectrum for Gadofluorine P as determined *in vitro*, right panel shows the theoretical spectrum together with the structure of Gadofluorine P. (**C**) MALDI-MSI of the aorta of the animal shown in (**A**) reveals the accumulation of Gd in plaques. (**D**) Elastica van Gieson staining of the same section shown in (**C**) and an overlay of MALDI-MSI and histology (**E**). (**F**) Upon magnification Gadofluorine P accumulation can be co-localized to plaque areas containing extra-cellular matrix (black arrows)(**F:** upper panel Elastica van Gieson staining, middle panel MALDI MSI, bottom panel overlay).

To verify Gadofluorine P accumulation in atherosclerotic plaques, we next performed MALDI MSI on the murine aortas, as described. Aortas were immediately excised after MR imaging. By using this approach, we were able to correlate the MR signal in atherosclerotic plaques, especially T1/R1 values (Fig. 2A), to the Gadofluorine P accumulation on MALDI images. Gadofluorine P can be detected by MALDI based upon its’ mass peak at *m/z* 1323 (M + H^+^, Fig. 2B). Gadofluorine P was indeed detected in atherosclerotic lesions which exhibited increased signal intensities on MR imaging (representative MALDI image in Fig. 2C). Subsequent histology was performed on the same tissue sections used for MALDI MSI. Corresponding Elastica van Gieson staining and overlay of histology and MALDI images of the same section show Gadofluorine P accumulation in atherosclerotic lesions especially of the aortic root (Fig. 2D,E). Magnification revealed that Gadofluorine P is present in ECM-rich regions of the atherosclerotic plaque (black arrows, Fig. 2F).

### Assessment of Gadofluorine P kinetics by mass spectrometry imaging

**Figure 3:**
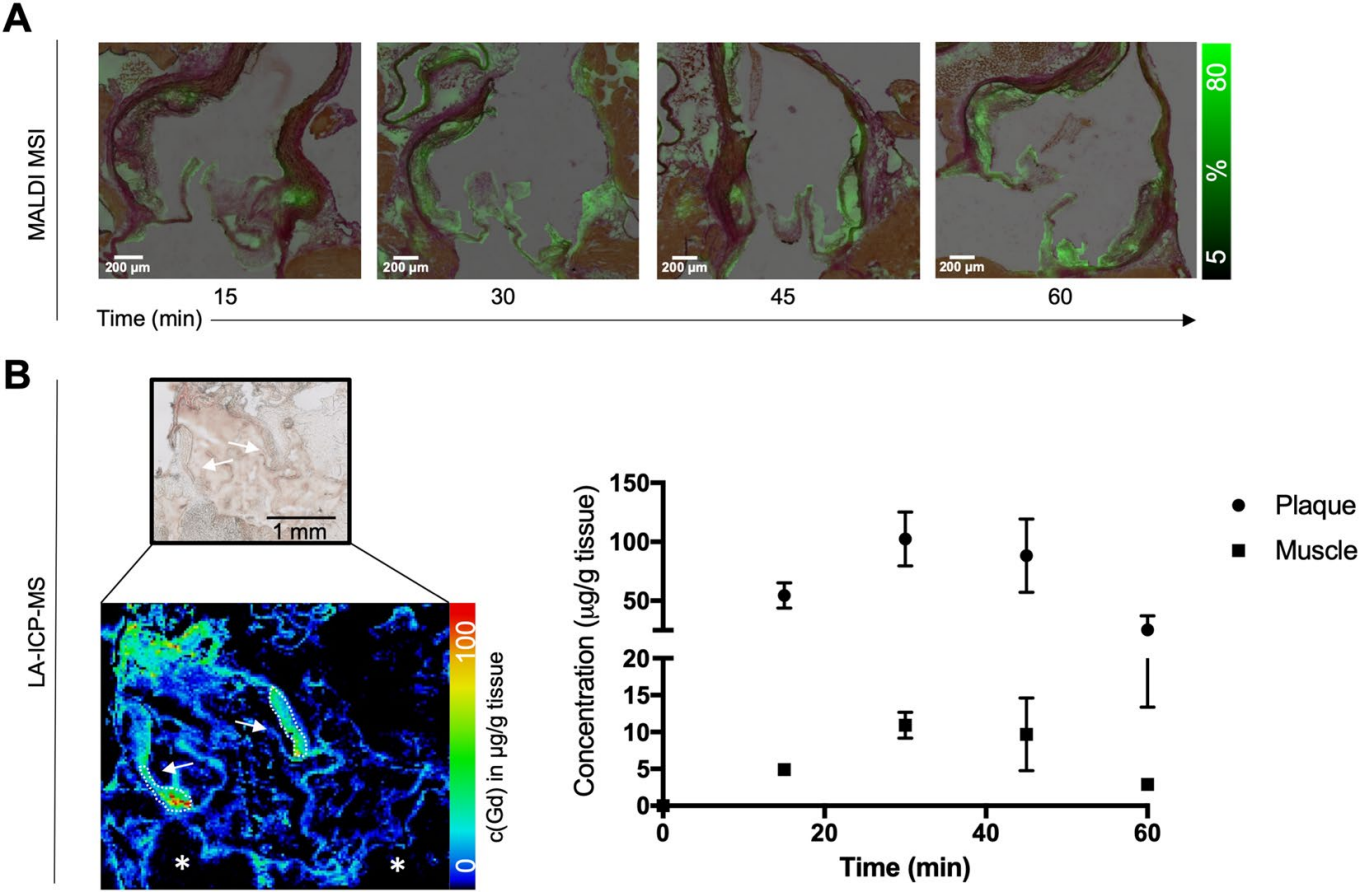
Determination Gadofluorine P kinetics by mass spectrometry imaging. (**A**) Gadofluorine P detection at baseline, 15, 30, 45 and 60 minutes following i.v. injection by MALDI MSI. Signal is shown as % of maximum intensity, not being fully quantitative. Representative images of one animal per time point is shown. Per time point n = 3 mice were investigated. (**B**) Elemental LA-ICP-MS of Gd(III) shows gadolinium accumulation at the aortic root (arrows) with a spatial resolution of 15 μm (top panel: white light microscopy image, bottom panel: LA-ICP-MS) and only minor Gd(III) accumulation in the adjacent myocardium (asterisk). Gd(III) concentrations peaked at 30 minutes post injection. Data shown as Mean ± SEM.

To verify Gadofluorine P kinetics by mass spectrometry imaging we performed MALDI with a spot size of 70 μm in aortas, which were excised at different time points after Gadofluorine P injection in atherosclerotic *Ldlr*^*−/−*^ mice after 16 weeks on high-fat diet (Fig. 3A). As recently demonstrated by Tang *et al*. it is required to use the same tissue type for concentration standards and for analysis to overcome tissue specific ion suppression effects^18^. Thus, an absolute quantitation of Gadofluorine P in mouse aortic plaques demands the application of concentration standards onto mouse aortic plaque sections. Due to the limited amount and size of aortic tissue, full quantification of Gadofluorine P in target tissue could not be achieved by MALDI.

We therefore continued to investigate absolute Gadofluorine P concentrations in the atherosclerotic wall by elemental laser ablation – inductively coupled plasma – Mass Spectrometry (LA-ICP-MS) which does not detect the entire Gadofluorine P molecule, but the elemental Gd. In LA-ICP-MS the entire sample is ablated, with the disadvantage that corresponding histology images can only be obtained from adjacent sections. However, the advantage of the full ablation is the quantitative character of the measurement. LA-ICP-MS with a spot size of 15 μm was performed from samples obtained at baseline, 15, 30, 45 and 60 minutes post injection (n = 3–6 per time point). At baseline (mice not injected with Gadofluorine P) the concentration of Gd was below the limit of quantification (LOQ = 197 ng/g, determined after the 10σ criterion). Gadofluorine P concentration (given in μg gadolinium / g tissue) within the atherosclerotic wall increased to 54.6 ± 10.7 μg/g (at 15 minutes), peaked with 102.4 ± 22.9 μg/g (at 30 minutes), and then decreased again to 88.4 ± 31.1 μg/g (at 45 minutes) and 25.3 ± 11.9 μg/g (at 60 minutes post injection). In contrast, Gd concentration in the adjacent myocardium remained below 10μg/g over the 60 minutes period (Fig. 3B).

### Analysis of Gadofluorine P accumulation during atherosclerosis progression

**Figure 4:**
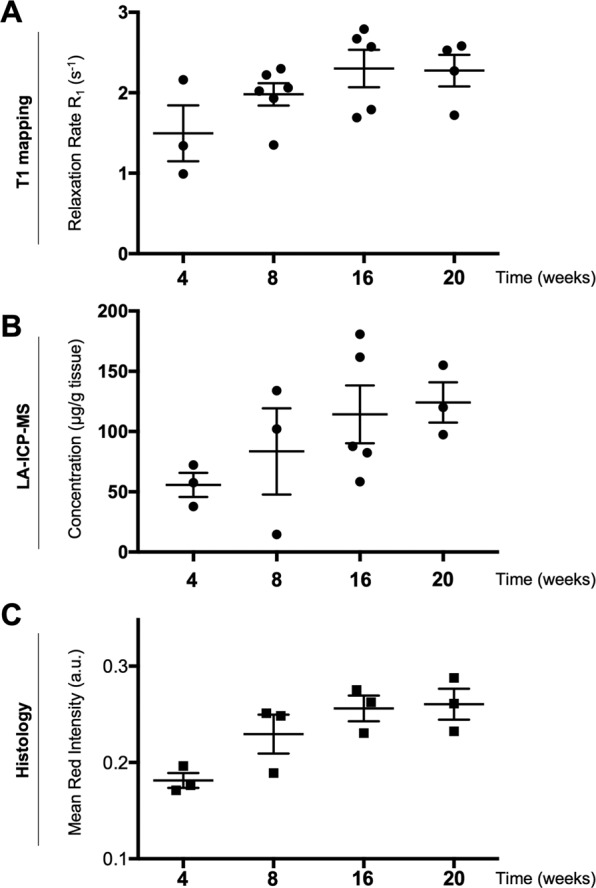
Monitoring of atherosclerosis formation by combined MR and mass spectrometry imaging. (**A**) R1 values from MR analyses of atherosclerotic lesions at the aortic root over the course of atherosclerotic plaque progression were corroborated by Gd(III) detection in corresponding lesions by LA-ICP-MS (**B**). The course of Gadofluorine P accumulation was accompanied by an increase in Mean Chromogen Red Intensity on Elastica van Gieson stains (**C**), indicative of increased ECM presence in atherosclerotic lesions. Scatter dot plots show Mean ± SEM, n = 3–6 mice per time point.

Having proven that mass spectrometry imaging, and in particular LA-ICP-MS serves as a complementary technique to verify gadolinium accumulation in tissue after *in vivo* MR imaging, we next thought to investigate the course of atherosclerosis progression by Gadofluorine P. Therefore, mice were investigated at 4, 8, 16 and 20 weeks after initiating the high-fat diet. MR imaging was performed, mice sacrificed immediately following imaging and further analyses by LA-ICP-MS and histology were performed. T1 mapping revealed increasing R1 values in atherosclerotic plaques until 20 weeks of high-fat diet (Fig. 4A), which were corroborated by increasing Gadolinium concentrations determined by LA-ICP-MS (Fig. 4B). To correlate the amount of Gadofluorine P accumulation with extracellular matrix (ECM) formation, mean chromogen red intensity was determined in atherosclerotic lesions as semi-quantitative measure of ECM. Analysis of Elastica van Gieson staining verified the course of Gadofluorine P accumulation by revealing increasing amounts of positive staining for ECM in atherosclerotic lesion (Fig. 4C).

**Figure 5:**
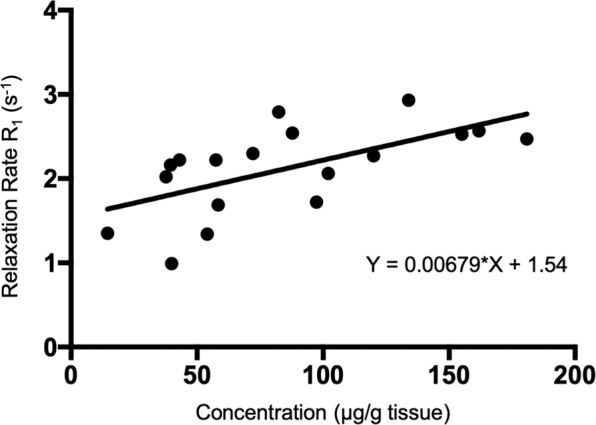
Conversion function. Gadofluorine P concentrations (from *ex vivo* LA-ICP-MS) and corresponding R1 values (obtained from *in vivo* MRI T1 mapping) of different time points of atherosclerosis formation (4–20 weeks of high-fat diet) allow calculation of a conversion function, which permits retrospective quantification of *in vivo* MR images.

To determine a relationship between MR signal generated by Gadofluorine P and actual Gd concentration at the target site, a conversion function for retrospective quantification was generated between corresponding MR images (T1 maps) and LA-ICP-MS images. As tissue deformation of the explanted aortic arch does not allow exact co-registration between MR and mass spec images we used maximum values for both signal intensity (R1) and maximum concentration in regions of interest at comparable anatomical locations. Maximum R1 values and maximum corresponding concentrations obtained at the different stages of atherosclerosis formation (4, 8, 16 and 20 weeks of high-fat diet) were plotted yielding an equation for the relationship between MR signal and its actual underlying substrate (Fig. 5). The resulting function demonstrates only moderate linearity between MR signal and concentration of gadolinium in tissue. In the future, this type of conversion function may allow the calculation of *in vivo* concentration maps calculated from T1 maps using the resulting equation. Thereby, the drawback of MRI as not fully quantitative imaging technique can be overcome and full quantitative values for *in vivo* molecular imaging probes can be obtained.

## Discussion

In the present study, we show that Gadofluorine P, an amphiphilic MR contrast agent, accumulates in atherosclerotic plaques and that Gadofluorine P accumulation increases during progression of atherosclerosis in *Ldlr*^*−/−*^ mice on a high-fat diet. We show that this experimental gadolinium-based MR probe can be visualized and quantified in atherosclerotic lesions by mass spectrometry imaging with a high spatial resolution down to 15 μm. Complementary bioimaging as a combination of molecular and elemental imaging techniques is a versatile tool for the visualization of Gadofluorine P. As MALDI-IMS better preserves tissue integrity of the sectioned specimen, an overlay of the MALDI image with the corresponding anatomical image obtained in histology can be obtained. This is not possible in LA-ICP-MS as the tissue gets substantially destroyed during the laser ablation process. Additionally, MALDI in general is able to generate multiplexed analyses, potentially allowing to investigate multiple target proteins of molecular gadolinium probes and the imaging probe itself in the same section in the future^19,20^. Moreover, MALDI is able to detect the entire and intact contrast agent molecule, while LA-ICP-MS reports elemental gadolinium, without any information if the gadolinium is still retained in the intact contrast agent molecule or maybe already released due to thermodynamic instability^21,22^. Taking advantage of the different strengths of the techniques like easy quantification and high spatial resolution for LA-ICP-MS and detecion of intact Gadofluorine P by MALDI leads to quantitative and comprehensive results. In terms of spatial resolution and quantification, LA-ICP-MS is clearly superior over MALDI-MSI. LA-ICP-MS provided a spatially-resolved quantification of gadolinium accumulation in atherosclerotic lesions of the aortic root which was in good agreement with the MR imaging signal, particularly the R1 values obtained from the T1 mapping sequences. Histology revealed that Gadofluorine P binding occurs in ECM-rich regions within the atherosclerotic plaque, which can be explained by its amphiphilic character. Both R1 values for Gadofluorine P (obtained *in vivo*) and corresponding concentrations determined by LA-ICP-MS (*ex vivo*) can be used to generate a conversion function which permits a retrospective quantification of *in vivo* imaging results^12^.

Quantification of the imaging signal is a prerequisite for precise analysis of plaque development and for monitoring pharmaceutical interventions of atherosclerosis. MR imaging has the advantage of providing morphologic, functional and molecular information. But at the same time MRI is semiquantitative only, with T1/R1 mapping being the most quantitative approach today. Relaxation rates depend on multiple parameters such as temperature, solvent, pH value and compartmentalization of the contrast agent. While in theory, relaxivity depends linearly on gadolinium concentration, metabolic processes may result in a nonlinear relationship between the MR signal and the gadolinium probe concentration^2^. Thus, there is a clear need for adjunct methodology to investigate the correlation between signal generation and the corresponding substrate. Visualizing gadolinium in tissue specimens has so far only been possible by transmission electron microscopy^3^ or after radioactive labeling of the gadolinium chelate^23^, which are not fully quantitative. Barkhausen *et al*. were the first who quantified gadolinium concentration in atherosclerotic plaques with ICP-MS. However ICP-MS requires homogenization of the entire specimen, leading to a loss of anatomical information^6^. Since mass spectrometry imaging has proven to depict gadolinium in tissue at high spatial resolution^9^, it is now possible to correlate probe concentration from the target site to the actual imaging signal. By adding mass spectrometry imaging, the source of the imaging signal can now be mapped quantitatively with high spatial resolution. Additionally, by computing a conversion function between the MR signal and probe concentration determined by LA-ICP-MS a retrospective quantification of the *in vivo* imaging can be obtained^12^. Within a similar experimental setting this equation function can then be used to derive quantitative values for Gadolinium content in tissue from corresponding T1/R1 values, without the need for sacrificing additional animals.

A remaining challenge of the approach presented here is that exact anatomical co-registration of mass spectrometry images and MR images is not possible until now. Firstly, slice thickness in MRI is much higher (in our study 1 mm) than that of mass spectrometry images (in our study 10μm). Secondly, the different slice thickness between MRI and mass spectrometry imaging has potential impact on the correlation function. Either a stack of MSI sections over the same thickness as the MR slice would have to be ablated or a single MSI section representative for the quantitative values of contained gadolinium has to be used. And third, harvesting of the aortic arch *ex vivo* for mass spectrometry imaging leads to an unavoidable tissue deformation. This subsequently leads to errors when creating overlays of both MRI and MSI images. Techniques of deformable image registration however may allow to co-register both datasets in the future^24^.

The present approach can be transferred also to other molecular MR contrast agents^9^. Gadofluorine M, the precursor compound of Gadofluorine P, has been shown to bind to atherosclerotic plaque components, both the lipid core and the altered ECM (particularly collagen, tenascin and proteoglycans). Targeting elastin, another very important ECM protein involved in atherosclerosis remodeling with a dedicated MR probe, has been shown to reliably report on atherosclerotic plaque burden by magnetic resonance imaging^3,5^. Successful plaque imaging with collagen-specific MR agents has been reported as well^25,26^.

In summary we show that Gadofluorine P accumulation in murine atherosclerotic lesions can be detected by *in vivo* molecular MRI and *ex vivo* mass spectrometry imaging. Mass spectrometry imaging thereby provides a spatially resolved quantification of the gadolinium probe allowing to retrospectively improve quantification of the *in vivo* imaging data. Deriving quantitative values from biomedical imaging is especially desired, when a process is tracked *in vivo* over time or if the response to therapies needs to be assessed.

## Methods

### Animal model

All animal procedures were approved by the animal ethic commission of the local authorities (Regierung von Oberbayern und Regierung von Unterfranken) and carried out in accordance with the Guide for the care and Use of Laboratory Animals (NIH, 2011). Animals were housed in standard animal rooms (12 h light/dark cycle, 50–60% humidity, 18 °C-23 °C temperature, bedding material) in individually ventilated cage systems (IVC Techniplast) under specific pathogen-free conditions with free access to water and standard laboratory chow *ad libitum*. A total of 48 mice was used for the study with group sizes of n = 3–6 mice per time point. Atherosclerosis was induced in female *Ldlr*^*−/−*^ mice (10 weeks old; Jackson Laboratory, USA) by feeding a high fat diet (HFD) for up to 20 weeks. *In vivo* imaging was performed at 4, 8, 16 and 20 weeks after initiating HFD (n = 6). For imaging procedures mice were deeply anesthetized by inhalation anesthesia (isoflurane 1.5–2.5% vol and 0.9 L O_2_). For assessment of the kinetics of Gadofluorine P accumulation in the atherosclerotic wall by *in vivo* MRI n = 3 mice were investigated longitudinally by MRI. Additionally, specimens of n = 3 mice for each time point (baseline, 15, 30, 45 and 60 minutes post injection) were investigated by post mortem MALDI-MSI and LA-ICP-MS after i.v. injection of 0.2 mmol/kg body weight of contrast agent. All mice were sacrificed by an injectable anesthetic overdose of ketamine-xylazine and the aortas were excised, snap frozen and stored at −80 °C until further analysis.

### Contrast agent

Gadofluorine P (invivoContrast GmbH, Berlin, Germany) is an amphiphilic, isoosmolar gadolinium agent with a molecular weight of 1322 Da. It is a derivative of Gadofluorine M, an experimental MR agent successfully applied for experimental MR imaging of atherosclerosis in rats, rabbits and swine^6,23,27–29^. Gadofluorine M exhibits a strong binding to plasma proteins such as albumin and thereby can be used to assess endothelial leakage, an early feature of atherosclerosis. Additionally, Gadofluorine M has been shown to target to the lipid core as well as to proteins involved in extracellular remodeling of the plaque such as collagen, proteoglycans and tenascin due to its amphiphilic character. To optimize biodistribution Gadofluorine M has been modified to Gadofluorine P. Plasma protein binding of Gadofluorine P is >90%, but shows a reduced plasma half-life in rats of about 2 hours.

### MR Imaging

Magnetic Resonance Imaging was performed on a horizontal bore 7 T small animal scanner (Disovery MR901, GE Healthcare, Chalfont St. Giles, United Kingdom) equipped with a 300 mT/m standard high-field gradient system and a 72 mm inner diameter ^1 ^H/^13 ^C quadrature birdcage resonator (Rapid Biomedical, Rimpar, Germany). The birdcage resonator was only used for transmission while signals were received by a 2-channel surface coil placed around the heart. Image acquisition was performed under free-breathing conditions and with prospective ECG triggering using a small animal monitoring and gating system (Rapid Biomedical, Rimpar, Germany). Electrodes for detection of the ECG signal were placed at the front paws. During imaging the animal core temperature was maintained at 38 °C using an MR-compatible air-heating system. After acquisition of standard scout scans, slices were planned in both oblique orientation in line with the aortic arch as for black blood imaging, LGE imaging and T1 mapping as well as axial slice orientation for T1 mapping. T1 is a quantification of the longitudinal relaxation time, which is shortened by gadolinium-based contrast agents. For ease of comparison the quantification of T1 mapping is frequently done by reporting the R1 relaxation rates, where R1 = 1/T1. Gadofluorine P was injected at a dose of 0.2 mmol/kg body weight via the tail vein. First, kinetic assessment of Gadofluorine P deposition within the atherosclerotic wall was performed to determine time point of maximum peak enhancement. For subsequent experiments, *in vivo* MR imaging was performed at indicated time points of western diet, at 30 minutes after contrast agent injection, the time point of peak enhancement in the vascular wall.

After selecting the suitable imaging planes for black blood imaging a double inversion recovery spin echo sequence was performed using the following parameters: FOV 30 × 30 mm, matrix 320 × 320 (in-plane resolution 94 µm), slice thickness 1 mm, TR/TE 271.5 ms/ 5.8 ms, flip angle 90°, 4 lines/RR interval, 8 scans. Inversion time 140 ms. For LGE assessment, a segmented inversion-recovery fast gradient echo sequence was performed using the following parameters: FOV 30 × 30 mm, matrix 192 × 192 (in-plane resolution 156 µm), slice thickness 1 mm, TR/TE 7.6 ms/ 3.1 ms, flip angle 60°, 4 lines/RR interval, 2 scans. Delay after inversion was set to 350 ms. Cardiac gating was set to give an effective TR of 1 s from one inversion to the next.

After black blood imaging acquisition single-slice T1 mapping was performed in the same slice geometry in line with the aortic arch. A standard inversion prepared prospectively ECG-gated CINE Look-Locker sequence was used. Immediately after the ECG-trigger signal, it applies a non-selective adiabatic inversion pulse which is followed by a fast spoiled gradient echo segmented k-space acquisition. Depending on the heart rate 2–3 RR intervals were used for signal acquisition while 2–3 intervals were left for T1 relaxation leading to an effective TR of ca. 3 s from one inversion to the next. Specific sequence parameters are as follows: FOV 30 × 30 mm, matrix 128 × 128 (in-plane resolution 234 µm), slice thickness 1 mm, TR/TE 5.1 ms/ 1.3 ms, flip angle 8°, 4 lines/RR interval, 4 scans.

For T1 estimation of Look-Locker data analysis was performed using an open-source software tool for the generation of relaxation time maps in MRI^30^. T1 maps were calculated from DICOM source images based on a 3-parameter Levenberg-Marquardt curve fitting procedure with a correction for read-out-induced attenuation of the relaxation curve as described.

### MALDI-MSI

Sectioning of fresh frozen aortas was performed as described previously^9^. For preparation of small-sized aortic plaque tissue, serial sectioning was performed cautiously. Frozen tissue samples were attached on sample stages by ice. Cryosections of 12 µm thickness were prepared using a CM1950 cryostat (Leica Microsystems, Wetzlar, Germany) and thaw-mounted onto precooled (−20 °C) conductive indium-tin-oxide (ITO) glass slides (Bruker Daltonik GmbH, Bremen, Germany). In order to improve attachment of tissue sections during successive washing and staining procedures, conductive slides were pre-coated with 1:1 poly-*L*-lysine (Sigma-Aldrich, Taufkirchen, Germany) and 0.1% Nonidet P-40 (nonylphenoxylpolyethoxylethanol 40, Sigma-Aldrich, Taufkirchen, Germany)^31–33^. Detection of Gadofluorine P was optimized as described previously^31^. In brief, to estimate measurability and for establishing optimal instrument settings, Gadofluorine P was spotted onto untreated liver sections in decreasing concentrations. This procedure mimics *in vivo* treatment with the respective substance and proofed to be a reliable model for the estimation of tissue background and peak interference in the respective m/z range. Furthermore it allows the optimization of detection (instrument parameters) and if necessary the optimization of detection quality using different matrix substances or solvents prior to animal treatment.

To obtain digital images for co-registration, heart sections were dried at room temperature and scanned using a flatbed scanner. Matrix solution, composed of 7 g/l CHCA (Sigma-Aldrich, Taufkirchen, Germany) in 70% methanol and 0.2% trifluoroacetic acid (TFA, Applied Biosystems, Darmstadt, Germany) was applied using ImagePrep spray device (Bruker Daltonik GmbH, Bremen, Germany) according to the manufacturer’s standard protocol and instrument settings. MALDI TOF Imaging measurements were carried out at a spatial resolution of 70 µm using an Ultraflex III MALDI TOF mass spectrometer (Bruker Daltonik GmbH, Bremen, Germany) in positive reflector mode with a sampling rate of 1.0 GS/s. A total of 200 laser shots were accumulated for each position measured. For data generation, the software packages FlexImaging 4.0 and FlexControl 3.0 (Bruker Daltonik GmbH, Bremen, Germany) were used. Image creation was performed using the monoisotopic [M + H]^+^ peak at *m*/*z* 1323.

After mass spectrometry measurements, matrix was removed with 70% ethanol and sections were stained with Elastica van Gieson staining (Morphisto GmbH, Frankfurt am Main, Germany) according to the manufacturer´s protocol. This staining combines picric acid and acid fuchsin to visualize collagen and connective tissue. In resulting slides, cell nuclei appear black-brown, elastic fibers black, collagen red and muscle yellow. The stained tissue slides were scanned using a digital slide scanning system (Mirax Desk, Carl Zeiss MicroImaging GmbH, Jena, Germany). Images were imported to the FlexImaging 4.0 software (Bruker Daltonik GmbH, Bremen, Germany) and merged with the mass spectrometry datasets.

MALDI Imaging experiments were evaluated visually using the FlexImaging 4.0 software (Bruker Daltonik GmbH, Bremen, Germany).

### LA-ICP-MS

For bioimaging analysis by means of LA-ICP-MS cryosections have to be prepared. The samples were fixed with TissueTek® on a cryoholder and 10 µm thick cryosections were prepared with a cryomicrotome model CRYOSTAR NX70 (ThermoFisher Scientific, Bremen, Germany). Generated sections were mounted onto glass slides (VWR, Darmstadt, Germany). For a quantitative analysis, external calibration was performed by using matrix-matched standards based on gelatin prepared as described previously^11^.

To validate the Gd concentration of the matrix-matched standards, total Gd concentration was determined after acidic digestion with ICP-MS. Digestion was performed by dissolving 50 mg of gelatin standard in HNO_3_ (2% v/v)^34^. External calibration was carried out by diluting certified Gd ICP-MS standard solution (1000 mg/L Gd, Sigma-Aldrich, Taufkirchen, Germany) in a range between 5 and 30 µg/L. To check plasma stability, Ho (1000 mg/L Ho, SCP Science, Quebec, Canada) was added to calibration and standard solutions in a final concentration of 1 µg/L. For analysis, the ICP-MS 2030 (Shimadzu, Kyoto, Japan) with a MiniTorch, Ni sampler and skimmer and a cyclonic spray chamber in combination with an ASX 560 autosampler (Teledyne Technologies, Thousand Oaks, CA, USA), was used. Analysis was performed in kinetic energy discrimination (KED) mode with Helium as collision gas. For bioimaging analysis, the previously described ICP-MS was coupled via Tygon tubing to a laser ablation system LSX-213 G2^+^ laser ablation system (Teledyne CETAC, Thousand Oaks, USA) including a HelEx II ablation cell. Helium was used as transport gas and an additionally introduced liquid (HNO_3_, 2% v/v) was used to create stable wet plasma conditions.

For the samples as well as for the standards a spot size of 15 µm was used and scan speed and laser energy were optimized to quantitative ablation. The quantification was based on the ^158^Gd isotope. For the standards, 10 lines in the middle of the standard section were analyzed and averaged intensities were used together with determined Gd concentrations to generate linear calibration (see Supplementary Figure 1 for exemplary calibration curve). Relative standard deviation between the lines of every standard were <10%, showing sufficient homogeneity in the middle of the cryosection used for analysis.

## Data analysis

Data are presented as mean value and standard error. Gadofluorine P accumulation in different compartments (blood, plaque, myocardium) over time was compared by two-way ANOVA, followed by Tukey’s test for multiple comparisons (GraphPad Prism Version 7.0b). A p-value < 0.05 was considered statistically significant.

## Supplementary information

Supplementary Figure.
