# Supplementary material for: Mass Spectrometry Imaging of atherosclerosis-affine Gadofluorine following Magnetic Resonance Imaging
Source: Sci Rep. 2020 Jan 9;10:79. doi: 10.1038/s41598-019-57075-6 (PMC6952459; doi:10.1038/s41598-019-57075-6)
Supplement: Supplementary file 1 — Supplementary Figure. [file 41598_2019_57075_MOESM1_ESM.pdf]

# Mass Spectrometry Imaging of atherosclerosis-affine Gadofluorine following Magnetic Resonance Imaging

Fabian Lohöfer*, Rebecca Buchholz*, Almut Glinzer, Katharina Huber, Helena Haas, Georgios Kaissis, Annette Feuchtinger, Michaela Aichler, Peter B. Sporns, Carsten Höltke, Miriam Stölting, Franz Schilling, René M. Botnar, Melanie A. Kimm, Cornelius Faber, Axel K. Walch, Alma Zernecke, Uwe Karst, Moritz Wildgruber

*equal contribution

Supplementary Figure 1

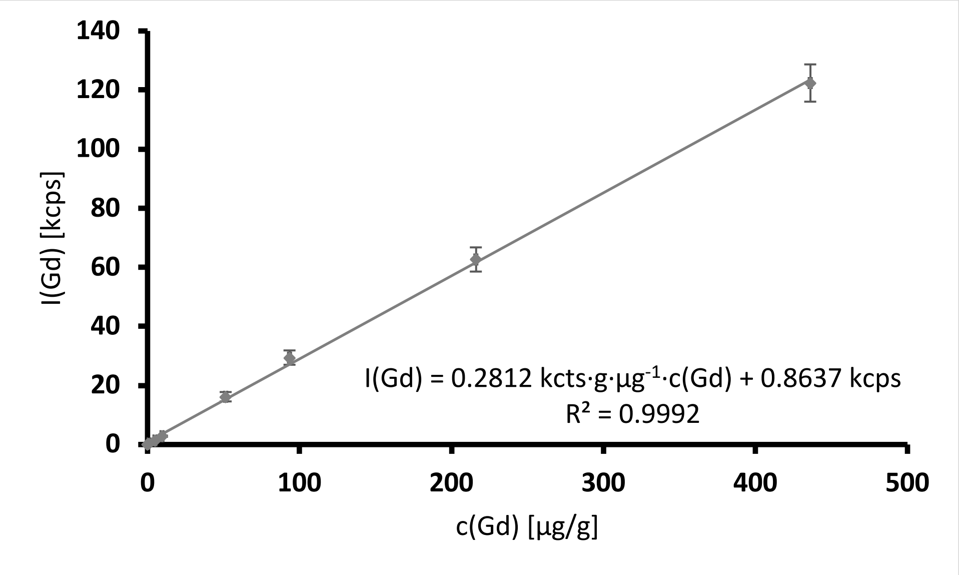


**Supplementary Figure 1.** Exemplary calibration curve for LA-ICP-MS imaging of Gadofluorine P

Calibration curve including linear calibration function and standard deviation. Intensities [kcps] were determined by LA-ICP-MS analysis of matrix-matched standards based on gelatin, concentrations [µg/g] were validated after acidic digestion with ICP-MS.
